# Assessing the robustness of AI lesion risk scores at different exposure settings using an anthropomorphic breast phantom

**DOI:** 10.1093/rpd/ncaf166

**Published:** 2026-03-13

**Authors:** L Alström, A Bjerkén, V Dahlblom, P Timberg, M Dustler, A Tingberg

**Affiliations:** Medical Radiation Physics, Department of Translational Medicine, Lund University, Malmö 205 02, Sweden; Radiation Physics, Department of Haematology, Oncology and Radiation Physics, Skåne University Hospital, Malmö 205 02, Sweden; Medical Radiation Physics, Department of Translational Medicine, Lund University, Malmö 205 02, Sweden; Radiation Physics, Department of Haematology, Oncology and Radiation Physics, Skåne University Hospital, Malmö 205 02, Sweden; Diagnostic Radiology, Department of Translational Medicine, Lund University, Malmö 205 02, Sweden; Department of Medical Imaging and Physiology, Skåne University Hospital, Malmö 205 02, Sweden; Medical Radiation Physics, Department of Translational Medicine, Lund University, Malmö 205 02, Sweden; Radiation Physics, Department of Haematology, Oncology and Radiation Physics, Skåne University Hospital, Malmö 205 02, Sweden; Diagnostic Radiology, Department of Translational Medicine, Lund University, Malmö 205 02, Sweden; Medical Radiation Physics, Department of Translational Medicine, Lund University, Malmö 205 02, Sweden; Radiation Physics, Department of Haematology, Oncology and Radiation Physics, Skåne University Hospital, Malmö 205 02, Sweden

## Abstract

To assess the robustness of risk scores provided by an artificial intelligence (AI) system for digital mammography (DM), when varying the exposure conditions. An anthropomorphic breast phantom containing a lesion, was imaged with DM at different tube voltages (kV), tube loadings (mAs), and anode/filter combinations (W/Rh, Mo/Mo, and Mo/Rh). The organ doses were extracted from the DICOM header and used as a substitute for average glandular dose. The images were analyzed with an AI system, which provided a lesion risk score which translates to suspicion for malignancy. Correlations between the lesion risk score and the exposure conditions were investigated. In most imaging conditions, weak to moderately strong positive associations between lesion risk scores and kV and mAs, respectively, were reported (varying by anode/filter combinations). When organ dose increased the AI risk scores plateaued, and further increase did not increase the lesion risk score. For typical clinical settings (W/Rh, 27 kV and 71 mAs) the range of lesion risk scores was 33–56 (mean: 42, SD: 9). Greatest reported variability (range: 36–63, mean: 51, SD: 12) was found at 27 kV and 36 mAs (using W/Rh). Images of suboptimal quality may result in inaccurate AI system performance. The unexpectedly large intra-group variability of AI risk scores should be further investigated.

## Introduction

Major advances in machine learning have led to an increased interest in implementation of artificial intelligence (AI) systems in the mammography workflow. Possible implementations of AI include acting as a decision support tool during double-reading, an independent second reader or a stand-alone reader to identify low-risk cases which then can be excluded from the double-reading process [1–6]. Research papers suggest that it is possible to reduce the number of false-positive recalls, increase the cancer detection rate and at the same time reduce the screen-reading workload of radiologists [1, 3, 4, 7, 8]. The benefits of integrating AI in the mammographic workflow have been demonstrated repeatedly. Some traditionally double-reading screening programs, such as Stockholm, Sweden [6], and Copenhagen, Denmark [9], have changed to using a single-reading strategy where the radiologist has access to AI. Experiences from Stockholm, where workflow is implemented as part of an ongoing project since June 2023, indicate that this has led to higher breast cancer detection rate and reduced workload for the radiologists [6]. Nevertheless, widespread adoption is hampered by breast radiologists’ lack of trust in AI systems and lack of prospective evaluation [10–12]. As AI tools move rapidly toward routine clinical use, it is important to validate AI systems intended for clinical use, assuring sufficient quality according to the current standard of mammography screening.

The process of validating an AI system includes the understanding of its possible limitations. Questions e.g. concerning AI systems’ responses to mammograms from various imaging modalities and variations in image quality need further investigation. This study aims at assessing the robustness of an AI system’s response to various image acquisition conditions. Images of an anthropomorphic breast phantom, acquired when varying exposure parameters, were analyzed by an AI system. Possible relationships between exposure parameters and risk scores were investigated.

## Material and methods

### Anthropomorphic breast phantom

**Figure 1:**
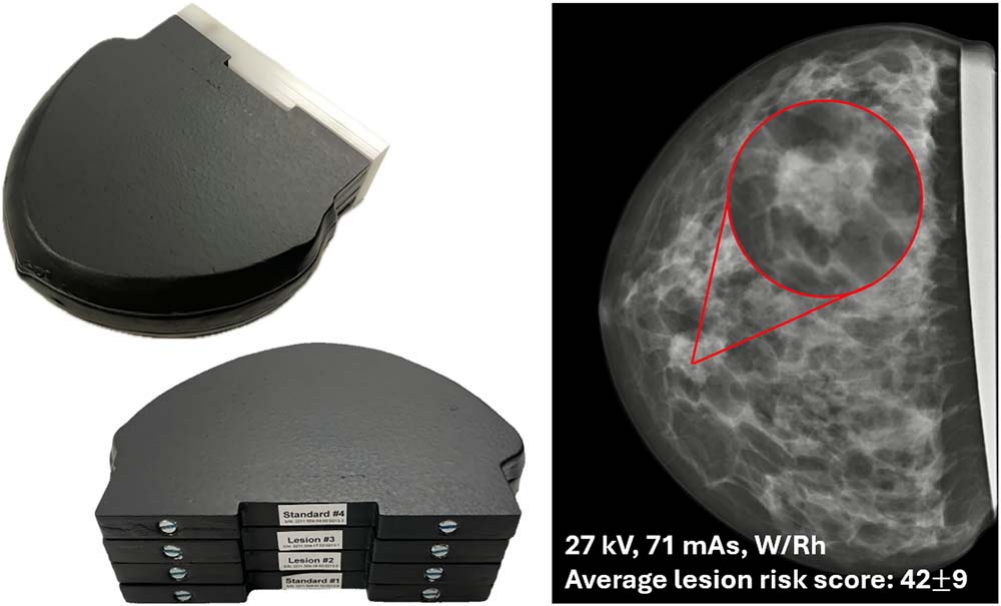
A 3D anthropomorphic breast phantom from PhantomX containing a lesion in the two central slabs, marked by the red circle in the X-ray image. The X-ray image is acquired with 27 kV, 71 mAs and W/Rh, corresponding to clinically obtained settings when using AEC. Average lesion risk score is 42$\pm$9 ($\pm$SD).

A 3D anthropomorphic breast phantom (59–01, PhantomX GmBH, Berlin, Germany) was used in this study (Fig. 1). It simulates a 36 mm compressed breast in the CC-view and consists of four slabs, containing realistic anatomical structures, such as adipose and glandular tissue, and has attenuation properties similar to that of a real breast [13–15]. For this project, the two central slabs were replaced with two slabs containing a spiculated mass of approximately 16 mm x 16 mm x 17 mm to represent a lesion. Breast radiologists at our hospital considered the appearance and characteristics of the tissue structure of the phantom to be highly relevant for evaluating the image quality of mammographic images. The spiculated mass was deemed highly suspicious for malignancy and would have caused a recall for further work-up in a clinical setting.

### Artificial intelligence system

The major reason for the advancements of available AI systems in mammography screening is the accessibility of incredibly large amount of data. With the introduction of screening programs, a large number of digital mammography (DM) examinations have been performed and for which the radiologist assessment in each case is known. This creates a suitable training database for deep convolutional neural networks, as the performance of an AI system always is limited by the available training data.

One of the commercially available AI systems for analyzing DM images is Transpara (version 2.1.0, ScreenPoint Medical, Nijmegen, Netherlands). Transpara is designed for automated breast cancer detection [4]. Information from the four standard views, CC and MLO of each breast, is analyzed and taken into consideration in the risk prediction. Transpara uses an image analysis algorithm to search for microcalcifications and soft-tissue lesions in the breasts [4]. The possible findings are then combined to deduce suspicious regions of the breast. When a suspicious region is present, Transpara will assign a region score, ranging from 1–100, where 100 represents greatest suspicion of cancer presence. The recorded finding with highest region score from all collected image views is used as input to a proprietary look-up-table, providing an overall exam score (Personal communication, A Rodríguez-Ruiz, 4 Nov 2025). The exam score spans from 1–10, where 10 indicates highest probability of cancer presence [4]. For clinical use, the exam scores are further divided into risk categories (low, intermediate, and elevated). However, for this study, only the region scores will be used.

### Image acquisition

DM images of the breast phantom were acquired using Siemens MAMMOMAT Inspiration (Siemens Healthineers, Erlangen, Germany). Image acquisition was done both with automatic exposure control (AEC) and with manually selected exposure settings where tube voltage (kV), tube loading (mAs), and anode/filter combination were varied. The images collected with AEC were used as a baseline as the exposure settings were varied relative to them. Based on the AEC settings, four different tube voltages ranging from the lowest to the highest were used (23, 27, 31, and 35 kV). For each level of tube voltage, the tube loading was varied in six steps, ranging from low to high exposures compared to AEC (10, 20, 36, 71, 140, and 280 mAs). Imaging at each combination of tube voltage and tube loading was repeated five times, by simply re-exposing the phantom. These imaging conditions were further repeated for each available anode/filter combination: W/Rh, Mo/Mo, and Mo/Rh.

For each DM image, the highest region score (for which the coordinates could be found within the visible tumor margin) was recorded. This region score was in this study referred to as the lesion risk score. If no finding in an image corresponded to the lesion, a lesion risk score of 0 was assigned to this image.

The organ doses were extracted from the DICOM headers and used as a substitute for the average glandular dose (AGD).

The post-processing parameters used were the ones that were used clinically in our hospital. These parameters were not varied in the current study.

### Data analysis

Descriptive statistics such as mean lesion risk scores and standard deviations (SD) were calculated for each data set, using Microsoft Excel.

For the varying exposure settings, the mean lesion risk scores, SD, and range were plotted together as a function of organ dose for each anode/filter combination. Plots were created using Python (version 3.12.2, Python Software Foundations, USA).

No results for signal-to-noise ratio (SNR) and contrast-to-noise ratio (CNR) were derived due to limitations of signal variations within regions of interest (ROIs) in a non-uniform image, as well as the effect of non-linearity of the image processing.

### Statistical analysis

Linear regression analysis was performed to isolate effects of mAs and kV, respectively, and investigate possible relationships between these parameters and lesion risk scores, for each anode/filter combination. kV was kept constant when lesion risk scores as a function of mAs (for each kV level) was investigated, and vice versa.

To test for statistical significance, the null hypothesis claiming that the linear regression coefficient, *B*, is equal to zero (*B* = 0) was assumed.

For all statistical tests performed, the obtained *p*-values were compared to a significance level of $\alpha$ = 0.05. The obtained *p*-values were not adjusted as each data point only was subjected to two hypothesis tests.

## Results

### Tungsten/Rhodium

Low mean lesion risk scores were observed for W/Rh at 23 kV and 10 or 20 mAs, and 27 kV at 10 mAs (Fig. 3). However, increased organ doses did not necessarily seem to yield higher lesion risk scores. The greatest reported variation of risk scores at repeated exposure with unchanged settings (only considering imaging conditions where a lesion risk score was presented in all five repeated exposures) was found at 27 kV and 36 mAs. The mean lesion risk score was 51 with SD of 12.

Significant associations could be observed between lesion risk scores and mAs at 23, 31, and 35 kV for the W/Rh images (Table 1). Despite this, the coefficients of determination indicated that the relationships were rather weak to moderate, suggested that little to some of the variances in the dependent variable were explained by the linear regression model. In case of constant mAs, significant associations could be observed between lesion risk scores and kV when 10, 20, and 280 mAs were used. These regressions were represented by higher coefficients of determination, indicated moderate to strong relationships where the regression model seemed to better explain the variances in lesion risk scores.

**Table 1:** Summary of univariate linear regression analysis of lesion risk scores at varied exposure settings for the W/Rh images. Linear regression coefficients (*B*) were calculated when kV and mAs, respectively, were constant to investigate the relationship between risk scores and one exposure setting at a time. The *P*-values associated with the regression coefficients are also stated, together with the coefficient of determination (*R*^2^).

Imaging condition		*B* (95% CI)	*P*-value	*R* ^2^
Constant kV, varying mAs	23 kV	0.105^*^ (0.049, 0.162)	<.001	0.342
	27 kV	0.035 (−0.014, 0.083)	.153	0.071
	31 kV	0.032^*^ (0.001, 0.062)	.041	0.140
	35 kV	0.040^*^ (0.020, 0.059)	<.001	0.380
Constant mAs, varying kV	10 mAs	2.470^*^ (1.454, 3.486)	<.001	0.592
	20 mAs	2.435^*^ (1.214, 3.656)	<.001	0.494
	36 mAs	0.535 (−0.505, 1.575)	.294	0.061
	71 mAs	−0.230 (−0.852, 0.392)	.448	0.032
	140 mAs	0.045 (−0.645, 0.735)	.892	0.001
	280 mAs	0.745^*^ (0.194, 1.296)	.011	0.310

^*^Relationship is significant at the 0.05 level.

**Figure 2:**
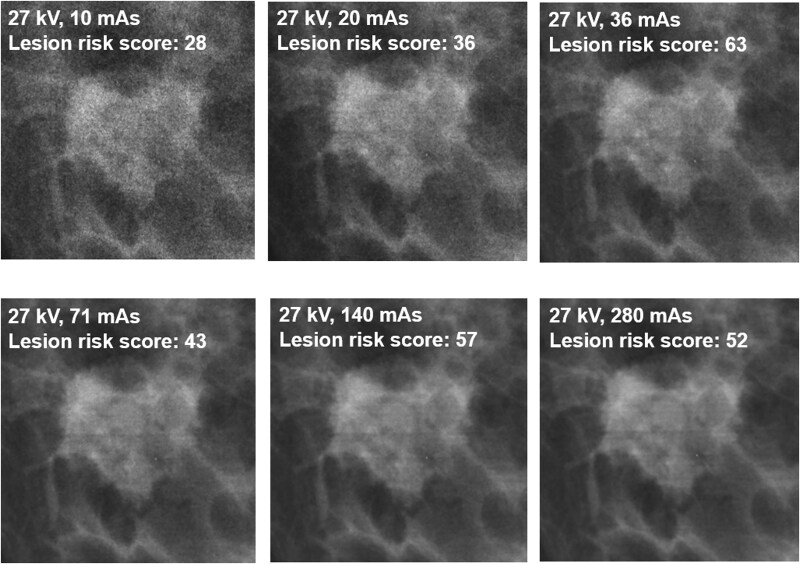
An image series illustrating the appearance of the lesion at 27 kV at various tube loadings (using W/Rh as anode/filter combination). The clinically obtained exposure settings (when using AEC) correspond to 27 kV and 71 mAs. The lesion risk score related to each image is also specified.

An image series for visual assessment of image quality at various tube loadings was included (Fig. 2). For the sake of interpreting the AI results, the images provide limited additional understanding of how the underlying algorithms relate to the variability in lesion risk scores. As illustrated, no obvious trend between presented lesion risk score and image quality is observed.

### Molybdenum/Molybdenum

Low mean lesion risk scores were reported at 23 kV and 10 or 20 mAs for Mo/Mo, similar to that obtained for W/Rh (Fig. 4). When visually examined, the distribution of lesion risk scores for Mo/Mo (compared to W/Rh) seemed to increase with increased organ dose up to a certain level, after which the lesion risk scores appeared to decrease. This effect was observed at organ dose levels far beyond what is clinically used. Generally higher organ doses were obtained, relative to that of W/Rh, despite the same combinations of mAs and kV. The greatest reported variation of risk scores at repeated exposure and unchanged settings was found at 31 kV and 36 mAs. The mean lesion risk score was 55 with SD of 13.

Significant associations could be observed between lesion risk scores and mAs at all kV levels for Mo/Mo (Table 2). The coefficients of determination indicated that the relationships were strong, except at 35 kV. In case of constant mAs, significant relationships could be observed between lesion risk scores and kV at all mAs levels except for 280 mAs. These linear fits indicated moderate to strong explanation of the variances in the dependent variable.

**Table 2:** Summary of univariate linear regression analysis of lesion risk scores at varied exposure settings for the Mo/Mo images. Linear regression coefficients (*B*) were calculated when kV and mAs, respectively, were constant to investigate the relationship between risk scores and one exposure setting at a time. The *P*-values associated with the regression coefficients are also stated, together with the coefficient of determination (*R*^2^).

Imaging condition		*B* (95% CI)	*P*-value	*R* ^2^
Constant kV, varying mAs	23 kV	0.202^*^ (0.138, 0.266)	<.001	0.591
	27 kV	0.121^*^ (0.088, 0.155)	<.001	0.665
	31 kV	0.133^*^ (0.092, 0.175)	<.001	0.606
	35 kV	0.050^*^ (0.000, 0.100)	.048	0.128
Constant mAs, varying kV	10 mAs	2.758^*^ (1.890, 3.627)	<.001	0.687
	20 mAs	3.300^*^ (1.964, 4.636)	<.001	0.599
	36 mAs	1.395^*^ (0.413, 2.377)	.008	0.331
	71 mAs	2.150^*^ (1.374, 2.926)	<.001	0.653
	140 mAs	1.465^*^ (0.715, 2.215)	<.001	0.483
	280 mAs	−0.345 (−1.398, 0.708)	.500	0.026

^*^Relationship is significant at the 0.05 level.

### Molybdenum/Rhodium

For Mo/Rh, the organ doses were generally higher (similar to Mo/Mo) compared to that of W/Rh, despite the same combinations of kV and mAs (Fig. 5). The low mean lesion risk scores observed at 23 kV and 10 mAs, 20 mAs or 36 mAs, and 27 kV with 10 mAs were similarly reported for W/Rh and Mo/Mo. When the distribution of lesion risk scores was visually checked, the scores seemed to increase with increased mAs for 23 kV. However, at 27 kV, 31 kV, and 35 kV, the lesion risk scores seemed to reach a plateau. This was also verified by the significant associations at 23 kV and varying mAs (Table 3). The coefficient of determination suggested a rather strong relationship. A weaker significant relationship was observed at 35 kV when mAs was varied.

**Table 3:** Summary of univariate linear regression analysis of lesion risk scores at varied exposure settings for the Mo/Rh images. Linear regression coefficients (*B*) were calculated when kV and mAs, respectively, were constant to investigate the relationship between risk scores and one exposure setting at a time. The *P*-values associated with the regression coefficients are also stated, together with the coefficient of determination (*R*^2^).

Imaging condition		*B* (95% CI)	*P*-value	*R* ^2^
Constant kV, varying mAs	23 kV	0.189^*^ (0.134, 0.245)	<.001	0.634
	27 kV	0.031 (0.000, 0.062)	.050	0.130
	31 kV	−0.002 (−0.020, 0.016)	.837	0.002
	35 kV	0.025^*^ (0.005, 0.044)	.015	0.195
Constant mAs, varying kV	10 mAs	2.965^*^ (1.805, 4.125)	<.001	0.616
	20 mAs	1.865^*^ (0.752, 2.978)	.002	0.408
	36 mAs	0.425 (−0.540, 1.390)	.367	0.045
	71 mAs	−0.120 (−0.710, 0.470)	.674	0.010
	140 mAs	−0.920^*^ (−1.483, −0.357)	.003	0.396
	280 mAs	−2.900^*^ (−3.734, −2.066)	<.001	0.813

^*^Relationship is significant at the 0.05 level.

In case of constant mAs, significant associations could be observed between lesion risk scores and kV at all mAs levels except for 36 and 71 mAs. At 140 and 280 mAs, the relationships between lesion risk scores and kV were represented by negative regression coefficients, indicated a decrease in lesion risk scores when kV was increased. At 280 mAs (and varying kV) the coefficient of determination suggested a strong negative relationship.

Additionally, the greatest reported spread of lesion risk scores for Mo/Rh at repeated exposure and unchanged settings was found at 31 kV and 10 mAs, with a mean lesion risk score of 38 and SD of 8.

## Discussion

This study evaluated the robustness of an AI system’s lesion risk scores on DM images acquired under varying exposure conditions across three anode/filter combinations. The system detected the lesion as a suspicious finding and provided a correctly localized score in all images acquired under AEC conditions and in >90% of images when tube voltage and tube loading were varied manually.

Linear regression analysis was chosen as statistical analysis method to investigate possible relationships between lesion risk scores and each exposure parameter, since it was not theoretically known how AI risk scores may differ with varying kV and mAs. However, it should be clarified that significant results not necessarily were interpreted as linear relationships between the dependent (lesion risk scores) and independent variables (kV and mAs). For that reason, linear regression coefficients were also reported to provide more information and avoid misinterpreting the results. Additionally, it should be mentioned that the obtained *P*-values were not adjusted as the purpose of this study was to search for possible correlations to further explore, rather than to confirm any established relationships.

**Figure 3:**
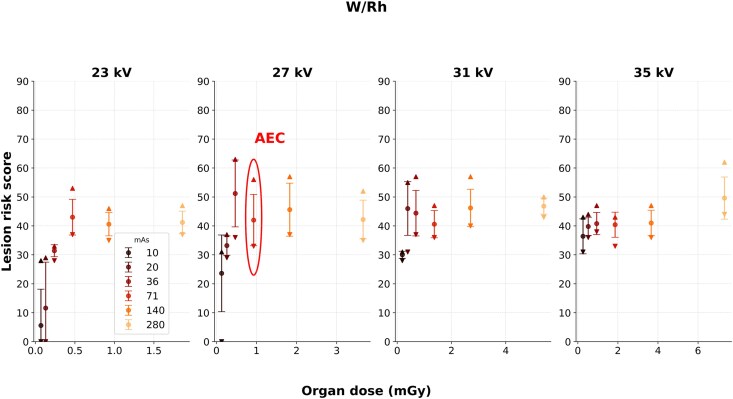
Lesion risk scores for W/Rh as a function of organ dose. The AEC settings corresponded to 27 kV and 71 mAs, resulting in an organ dose of 0.93 mGy. The data points and error bars indicate the means and SD, respectively. The upper and lower triangular markers represent the range of scores within each data set.

Significant associations, ranging from weak to moderately strong, were found between exposure parameters and lesion risk scores, though these varied by anode/filter combination. For W/Rh, visual inspection and regression results (Fig. 3, Table 1) showed no clear trends except at the lowest exposure settings (23 kV and 10 or 20 mAs, and 27 kV at 10 mAs). Low lesion risk scores were reported due to noisy images. For cases in which the AI failed to provide any score for the lesion, the images received default scores of 0, lowering the average lesion risk score. Although these low-dose conditions are not clinically relevant, they help demonstrate the AI system’s performance limits across a wide exposure range.

Notably, the largest intra-group variability in risk scores occurred near standard AEC settings (e.g. 27 kV, 36–71 mAs and W/Rh), where one would expect more consistent output due to training on clinically acquired images. This unexpected spread, encompassing multiple risk categories, raises questions regarding the system’s precision even under typical imaging conditions.

**Figure 4:**
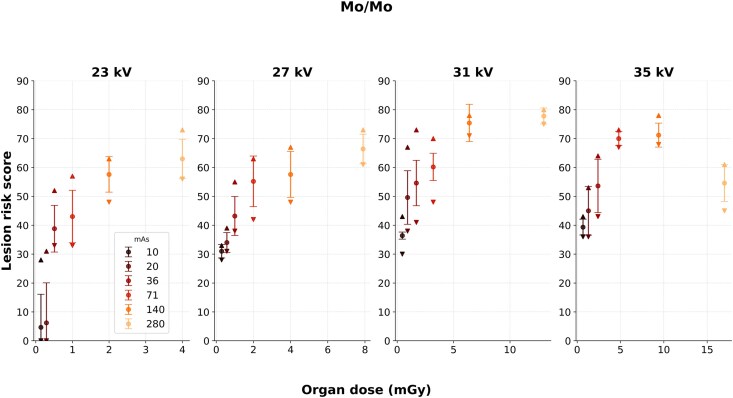
Lesion risk scores as a function of organ dose for the Mo/Mo images at varied kV and mAs levels. The settings closest corresponding to AEC was 27 kV and 71 mAs, resulting in an organ dose of 2.0 mGy. The data points and error bars indicate the means and SD, respectively. The upper and lower triangular markers represent the range of scores within each data set.

For Mo/Mo, low mean lesion risk scores were reported at 23 kV with 10 or 20 mAs for the same reasons as for W/Rh. The generally higher organ doses obtained, despite the same combinations of mAs and kV, indicated that the Mo/Mo X-ray spectrum was ‘softer’, contained more photons with lower energy compared to the X-ray spectrum of W/Rh. Mo/Mo demonstrated the most pronounced score changes in response to exposure variations (Fig. 4, Table 2), especially at 23 kV and 20 mAs. However, an unexplained drop in scores was seen at the highest kV and mAs settings, despite similarly high organ doses in Mo/Rh not producing such effects. For this reason, the coefficients of determinations did not indicate a strong association at 35 kV. Also, no significant relationship was reported when varying the kV at 280 mAs. The cause may relate to the AI system’s training range but warrants further investigation.

**Figure 5:**
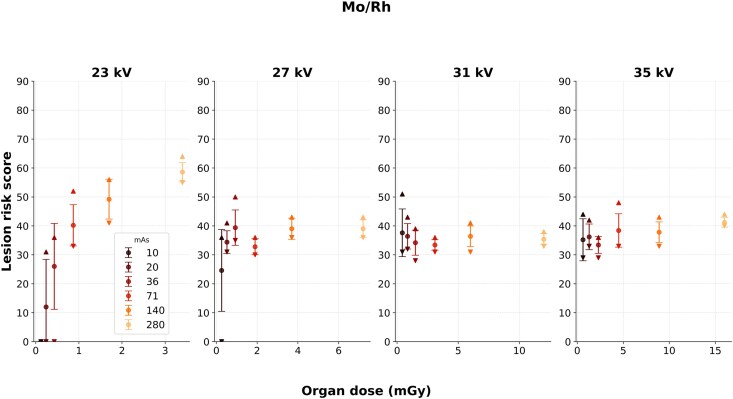
Lesion risk scores as a function of organ dose for the Mo/Rh images. The setting that closest corresponded to AEC was 27 kV and 71 mAs, resulting in an organ dose of 1.9 mGy. For 35 kV, the highest possible mAs was 256, even though 280 was chosen. The data points and error bars indicate the means and SD, respectively. The upper and lower triangular markers represent the range of scores within each data set.

Mo/Rh showed mixed results (Fig. 5, Table 3). Low mean lesion risk scores were also reported for Mo/Rh at low exposure settings, for the same reason as for W/Rh and Mo/Mo. A clear increase in scores was seen with rising mAs at 23 kV, while at higher mAs levels (140 and 280 mAs), increasing kV surprisingly reduced risk scores. This may reflect the system’s unfamiliarity with high-dose, high-kV conditions, and resulting image quality.

The widest score variation for Mo/Mo and Mo/Rh when Transpara successfully produced lesion risk scores for all five repeated images, was found at 31 kV with low or moderate mAs, potentially due to combined effects of lower contrast and reduced SNR complicating image analysis.

Comparisons between anode/filter combinations are challenging as the organ doses vary due to the different X-ray spectra, though efforts were made to standardize exposure variations relative to AEC values. While no formal statistical comparison was made between combinations, this limitation should be considered when interpreting inter-group differences.

To the authors’ knowledge, only one previous study—also by our group—has assessed AI performance across different exposure settings, reporting similar findings [16]. The current study is much more detailed compared to the previous study, which can be considered to be a pilot study. The interpretation of the two studies is essentially similar.

A key limitation is the use of only one mammography system, one AI product, and a breast phantom. Future studies should include multiple vendors and AI systems, as well as real patient images, to enhance generalizability.

## Conclusions

This study found, in most imaging conditions, weak to moderately strong positive associations between lesion risk scores and exposure parameters, suggesting that beam quality affects AI output. A threshold of image quality with respect to contrast and noise may exist, below which the system fails to perform reliably.

The most notable finding was the unexpectedly large variation in lesion risk scores across repeated exposures with identical settings. Whether this reflects limited precision of the AI system or study-related factors remains unclear and warrants further investigation. The consequences of this inconsistency of the AI system in a clinical setting remains to be studied.

## References

[1] Dahlblom V, Dustler M, Tingberg A. et al. Breast cancer screening with digital breast tomosynthesis: comparison of different reading strategies implementing artificial intelligence. Eur Radiol 2023;33:3754–65. 36502459 10.1007/s00330-022-09316-y PMC10121528

[2] Pacilè S, Lopez J, Chone P. et al. Improving breast cancer detection accuracy of mammography with the concurrent use of an artificial intelligence tool. Radiol Artif Intell 2020;2:e190208. 10.1148/ryai.2020190208 33937844 PMC8082372

[3] Lång K, Dustler M, Dahlblom V. et al. Identifying normal mammograms in a large screening population using artificial intelligence. Eur Radiol 2021;31:1687–92. 10.1007/s00330-020-07165-1 32876835 PMC7880910

[4] Rodriguez-Ruiz A, Krupinski E, Mordang JJ. et al. Detection of breast cancer with mammography: effect of an artificial intelligence support system. Radiology 2019;290:305–14. 10.1148/radiol.2018181371 30457482

[5] Rodriguez-Ruiz A, Lång K, Gubern-Merida A. et al. Can we reduce the workload of mammographic screening by automatic identification of normal exams with artificial intelligence? A feasibility study. Eur Radiol 2019;29:4825–32. 10.1007/s00330-019-06186-9 30993432 PMC6682851

[6] Dembrower K, Crippa A, Colón E. et al. Artificial intelligence for breast cancer detection in screening mammography in Sweden: a prospective, population-based, paired-reader, non-inferiority study. Lancet digit. Health. 2023;5:e703–11. 10.1016/S2589-7500(23)00153-X 37690911

[7] Hernström V, Josefsson V, Sartor H. et al. Screening performance and characteristics of breast cancer detected in the mammography screening with artificial intelligence trial (MASAI): a randomised, controlled, parallel-group, non-inferiority, single-blinded, screening accuracy study. Lancet Digit Health. 2025;7:e175–83. 10.1016/S2589-7500(24)00267-X 39904652

[8] Dahlblom V, Dustler M, Bolejko A. et al. Malmö breast ImaginG database: objectives and development. J Med Imaging (Bellingham) 2023;10:061402. 10.1117/1.JMI.10.6.061402 36779038 PMC9905220

[9] Lauritzen AD, Lillholm M, Lynge E. et al. Early indicators of the impact of using AI in mammography screening for breast cancer. Radiology. 2024;311:e232479. 10.1148/radiol.232479 38832880

[10] Shinners L, Aggar C, Grace S. et al. Exploring healthcare professionals' understanding and experiences of artificial intelligence technology use in the delivery of healthcare: an integrative review. Health Informatics J 2020;26:1225–36. 10.1177/1460458219874641 31566454

[11] Högberg C, Larsson S, Lång K. Engaging with artificial intelligence in mammography screening: Swedish breast radiologists’ views on trust, information and expertise. Digit Health 2024;10:20552076241287958. 10.1177/20552076241287958 39381821 PMC11459539

[12] Lee AY, Friedewald SM. Clinical implementation of AI in screening mammography: the essential role of prospective evaluation. Radiology 2024;311:e241124. 10.1148/radiol.241124 38832882

[13] Graff C . A new, open-source, multi-modality digital breast phantom. Medical imaging 2016: physics of medical. Imaging 2016;9783:978309-1-10.

[14] Ikejimba LC, Graff CG, Rosenthal S. et al. A novel physical anthropomorphic breast phantom for 2D and 3D x-ray imaging. Med Phys 2017;44:407–16. 10.1002/mp.12062 27992059

[15] Jahnke P, Schwarz S, Ziegert M. et al. Paper-based 3D printing of anthropomorphic CT phantoms: feasibility of two construction techniques. Eur Radiol 2019;29:1384–90. 10.1007/s00330-018-5654-1 30116957

[16] Tingberg A, Dahlblom V, Bakic PR. et al. AI lesion risk score at different exposure settings. Chicago: IL, USA, 2024. 10.1093/rpd/ncaf166 PMC13046041 41821441

